# Rapid cycling genomic selection in maize landraces: a step toward closing the yield gap

**DOI:** 10.1007/s00122-025-05107-3

**Published:** 2025-12-15

**Authors:** Carolina Rivera-Poulsen, Clara Polzer, Armin C. Hölker, Thomas Presterl, Sofia da Silva, Michelle Terán-Pineda, Milena Ouzunova, Albrecht E. Melchinger, Chris-Carolin Schön

**Affiliations:** 1 Plant Breeding, TUM School of Life Sciences, Technical University of Munich, 85354 Freising, Germany; 2 KWS SAAT SE & Co. KGaA, 37574 Einbeck, Germany; 3 Institute of Plant Breeding, Seed Science and Population Genetics, University of Hohenheim, 70593 Stuttgart, Germany; 4 Present Address: KWS SAAT SE & Co. KGaA, 37574 Einbeck, Germany; 5 Present Address: HZPC Research B.V., Roptawei 4, 9123 JB Metslawier, The Netherlands

## Abstract

**Key message:**

Rapid cycling genomic selection is a highly efficient tool for pre-breeding of maize landraces for complex traits, especially in combination with multi-trait selection and model retraining.

**Abstract:**

The introduction of landrace-derived material into modern breeding programs can only succeed if it performs satisfactorily for yield and other key agronomic traits. In this study, we explored the prospects of rapid cycling genomic selection in maize (*Zea mays* L.) to accelerate pre-breeding of landraces in comparison with recurrent phenotypic selection. We performed three cycles of genomic selection for testcross performance. The selection criterion was based on directional selection for biomass yield and stabilizing selection for plant height and flowering time. The prediction model was trained on testcrosses of 419 doubled-haploid (DH) lines derived from two European landraces. To estimate selection response and prediction accuracies, DH lines from all cycles (N = 204, C0–C3) were evaluated together with seven commercial hybrids in seven environments. Selection narrowed the yield gap to the commercial hybrids significantly with an increase in dry matter yield of about 10% in comparison with the reference population (C0). Despite stabilizing selection for plant height and flowering time, both traits showed a correlated response with biomass yield pointing to the importance of optimizing multi-trait selection, especially in landraces. Prediction accuracies were intermediate to high in the training population and decreased in the following cycles. Retraining the prediction model increased the prediction accuracy for all traits. Our results support the hypothesis that pre-breeding can be accelerated significantly by rapid cycling genomic selection and give valuable insights into key factors determining its success.

**Supplementary Information:**

The online version contains supplementary material available at 10.1007/s00122-025-05107-3.

## Introduction

Until the mid-twentieth century, cultivation of maize landraces was an integral part of agriculture in many countries of Western and Southern Europe (Reif et al. 2005). Introduced to Europe after discovery of the New World, maize rapidly adapted to a wide range of agro-climatic conditions in diverse regions through centuries of mild selection by farmers (Tenaillon and Charcosset 2011). This process led to the emergence of locally adapted, open-pollinated populations shaped by traditional cultivation practices and diverse end uses (Camacho Villa et al. 2005).

With the advent of hybrid maize breeding, this rich genetic heritage began to dwindle in the 1950s (Reif et al. 2005). The widespread adoption of hybrids, in Central Europe primarily based on the flint × dent heterotic pattern, marked a dramatic shift in breeding paradigms (Bunting 1968). Hybrids, selected for uniformity and high yield, rapidly replaced traditional landraces (Barrière et al. 2006). Importantly, only a limited number of landraces (e.g. Lacaune, Lizargarotte, Gelber Badischer, Rheintaler (Messmer et al. 1992)) were used to derive the first-cycle inbred lines for the European flint heterotic pool (Strigens et al. 2013). Moreover, only a small number of those lines contributed to the elite flint germplasm that has dominated breeding programs in Central Europe over the past seven decades (Balconi et al. 2024). This narrowing of the genetic base has raised concerns about genetic vulnerability (Brown 1983), particularly in the face of climate change (Zhao et al. 2017), evolving pests and diseases, and the plateauing of yield gains (Duvick 2005; Grassini et al. 2013; Zhang and Lu 2024).

Despite their marginalization, European landraces have not been lost entirely. Thanks to collection efforts initiated in the 1950s, over 5,000 accessions are nowadays conserved in genebanks across Europe (https://eurisco.ipk-gatersleben.de). These landraces are widely regarded as a “gold reserve” for future breeding progress (FAO 2010; Hoisington et al. 1999). Their known value as donors of major-effect alleles—especially for disease resistance and grain quality traits—is undisputed (Lazaridi et al. 2024). Yet, their utility for the improvement of complex, polygenic traits such as yield, drought tolerance, and resource use efficiency has remained underexplored (Strigens et al. 2013).

In hybrid breeding of allogamous crops like maize, a major barrier to exploit landrace diversity is the efficient development of fully homozygous inbred lines. The traditional approach of recurrent selfing reveals the genetic load inherent in landrace populations incrementally (Sood et al. 2014). This results often in very low efficiency of inbred line production or loss of potentially useful lines at the end. The introduction of the doubled-haploid (DH) technology has revolutionized this process, enabling the production of fully homozygous lines and efficient purging of deleterious alleles in a single step (Melchinger et al. 2017). However, DH line production from landraces requires about ten times the resources compared to elite germplasm (Strigens et al. 2013). Furthermore, some landraces are recalcitrant to the current method of haploid induction and genome doubling (Prigge et al. 2011).

The most significant hurdle in the utilization of landraces in breeding is their wide performance gap compared to elite germplasm that has been under intensive selection for decades (Böhm et al. 2017; Brauner et al. 2019; Hölker et al. 2019). In the pre-genomic era, the collaborative Latin American Maize Project (LAMP) phenotyped more than 12,000 native accessions of maize in a wide range of environments for many agronomic traits (Pollak 2003). The best LAMP accessions were selected for a bridging project (Germplasm Enhancement of Maize (GEM)) in which landraces were crossed with elite inbred lines. However, even after intense phenotypic selection in the progeny, a substantial performance gap remained in comparison to elite material (Salhuana and Pollak 2006). In addition, there is a high risk of losing favorable landrace alleles due to the linkage with detrimental alleles and/or unconscious selection in favor of the recurrent elite parent in backcross generations (Gorjanc et al. 2016).

The development of DH lines directly from landrace populations in combination with recurrent genomic selection offers an attractive alternative to phenotypic selection. Genomic selection allows to shorten breeding cycles by predicting breeding values from genome-wide marker data and has shown promise in increasing the efficiency of selection in population improvement (Crossa et al. 2017; Dreisigacker et al. 2023). Hölker et al. (2022) demonstrated that DH lines derived from maize landraces can serve as effective training sets for predicting both line per se and testcross performance despite a rapid decline of linkage disequilibrium over short genomic distances. More recently, Polzer et al. (2025) reported the successful implementation of rapid cycling genomic selection for early developmental traits in lines per se over three selection cycles.

While these findings are encouraging, comprehensive evidence on the selection response for testcross performance—particularly for key agronomic traits in maize such as yield, plant height, and maturity—has remained scarce. In this study, we propose the targeted improvement of a pre-selected landrace population by combining directional selection for biomass yield and stabilizing selection for plant height and female flowering as target traits. In addition to testcross performance, we also monitored line per se performance for early developmental traits as selection was initiated in the same base population that was used by Polzer et al. (2025).

The objectives of our study were to (i) quantify the selection response for testcross performance over three cycles of rapid cycling recurrent genomic selection of biomass yield, plant height, and female flowering; (ii) assess the yield and agronomic performance gap between commercial hybrids and testcrosses of the DH lines across selection cycles; (iii) evaluate the genomic prediction accuracy across selection cycles and the potential for improving it through model retraining with genotypes from advanced cycles; and (iv) monitor the molecular and genetic variance across selection cycles.

## Materials and methods

### Training set

Model training for all selection steps was based on a training set of 419 DH lines. These lines, subsequently referred to as data set PK, were derived from the maize landraces Petkuser Ferdinand Rot (PE, Germany, N = 203) and Kemater Landmais Gelb (KE, Austria, N = 216). All lines were genotyped with the 600 k single nucleotide polymorphism (SNP) Affymetrix® Axiom® Maize Array (Unterseer et al. 2014). The phenotypic diversity of the landrace-derived DH lines is illustrated in Fig. S1.

Phenotypic data for testcross performance used in model training were based on testcrosses of the 419 DH lines with the dent tester line F353. Details regarding the experimental setup and phenotypic data collection in four locations across Germany in 2018 were described in Hölker et al. (2019). Trials were repeated in the same locations in 2019, providing data from eight environments (4 locations × 2 years), covering a broad range of agro-climatic conditions. Briefly, in each environment the testcrosses of DH lines were planted together with 10 check hybrids in four adjacent lattice designs. Six agronomic traits were recorded (Table S1). Biomass yield (dt/ha), referring to the total whole-plant biomass harvested at silage stage, was calculated by multiplying the fresh matter yield by the dry matter content of the harvested material, determined from a subsample by weighing, oven-drying, and re-weighing. Additionally, dry matter content (%), plant height (cm), female flowering (days after sowing), and early plant height at growth stages V4 (cm) and V6 (cm) were recorded.

### Predictions

Genomic estimated breeding values (GEBVs) were calculated as detailed in Hölker et al. (2019) using the genomic best linear unbiased prediction (GBLUP) model

1 $${\boldsymbol{y}} = \boldsymbol{X\mu } + {\boldsymbol{Zu}} + {\boldsymbol{e}},$$

where ${\boldsymbol{y}}$ refers to the vector of adjusted entry means across environments for testcross performance of the $n$ = 419 DH lines from PK. ${\boldsymbol{\mu}}$ = (${\mu }_{j}$) is a vector of fixed effects for the landraces, with  *j* = 1, 2 representing PE and KE. ${\boldsymbol{u}}$ is an $n$-dimensional vector of the random genotypic effects for testcross performance of the DH lines, where ${\boldsymbol{u}}\boldsymbol{ }\sim N(0,\mathbf{K}{\sigma }_{g}^{2})$, with ${\sigma }_{g}^{2}$ being the genetic variance. $\mathbf{K}$ is the $n\times n$ realized genomic relationship matrix, calculated with method 1 of VanRaden (2008), based on allele frequencies of the population PE in PK from which the founders of the selection experiment were derived. ${\boldsymbol{e}}$ is a $n$-dimensional vector of random residuals, where ${\boldsymbol{e}} \sim N\left(0,\mathbf{I}{\sigma }^{2}\right)$ with $\mathbf{I}$ being an $n\times n$ identity matrix and ${\sigma }^{2}$ the error variance. ${\boldsymbol{X}}$ and ${\boldsymbol{Z}}$ represent incidence matrices, linking ${\boldsymbol{\mu}}$ and ${\boldsymbol{u}}$ with ${\boldsymbol{y}}$. GEBVs were calculated using the R package ASReml-R (Butler et al. 2009).

### Selection and recombination

The selection experiment focused on testcross performance of material derived exclusively from the 203 PE DH lines (subsequently denoted as population C0) to avoid confounding effects due to population structure. We applied (i) directional selection for increased biomass yield (BY) and (ii) stabilizing selection for plant height and female flowering to minimize undesired correlated responses. In all three selection cycles, a selection criterion integrating these three traits was used: (1) GEBVs were obtained from a multi-trait model (extended version of Eq. 1). (2) GEBVs were mean-centered and standardized by their standard deviation within each cycle. (3) GEBVs for plant height and female flowering were transformed by taking absolute values, PH_trans_ and FF_trans_, respectively. (4) The selection criterion for candidate $i$ was computed using the following formula:

2 $$SC_{i} = 2*BY_{i} - PH_{{trans_{i} }} - FF_{{trans_{i} }} .$$

The selection scheme depicted in Fig. S2 followed the approach outlined in our companion paper (Polzer et al. 2025). Selection was initiated by choosing the nine C0 lines (C0sel) with the highest values for the selection criterion (Eq. 2) as founders. These were crossed in a diallel mating design to produce 36 single-crosses (C1-S_0_ population), followed by self-pollination of the S_0_ plants to generate 1,030 S_1_ plants (C1-S_1_ population). The S_1_ plants were genotyped using an 11 k SNP array (see below), and their values for the selection criterion were computed based on GEBVs derived from their genomic relationship to the training set (VanRaden 2008).

Thirty-six C1-S_1_ plants (C1-S_1_sel) were selected based on the selection criterion and paired to produce 18 full-sib families, maximizing the modified Rogers’ distance [MRD, (Wright 1978)] between parents. The resulting 1,003 C2-S_0_ plants underwent the same genotyping and selection process as described for C1. Subsequently, the 32 selected plants (C2-S_0_sel) were arranged in 16 crossing pairs to establish the C3-S_0_ population. To ensure a sufficient effective population size ${N}_{e}$, the number of progenies from each C0sel founder line was limited in both cycles, with a maximum of 10 and 20 genotypes per founder in C2 and C3, respectively.

### Field experiments for assessing testcross and line per se performance

To assess the phenotypic response to recurrent genomic selection, DH lines were developed from the S_0_ populations of cycles C1 to C3 by in vivo haploid induction (Chaikam et al. 2019). DH lines from all cycles were used for (a) producing testcross seed with the dent inbred tester line F353 by manual crossing using the tester line as female parent to avoid variation in seed quality and (b) selfing for seed multiplication, required for line per se trials. Cycle C0, which is the base line for comparisons, was represented by random subsets of DH lines (C0r) in testcross and line per se trials. The samples C0r_TP_ and C0r_LP_ included few DH lines not represented in the training set and overlapped only partially due to limited testcross seed availability. On average, 50 and 78 DH lines per cycle were evaluated in testcross and line per se trials, respectively, with substantial overlap (Table S2). In total, 204 DH lines were evaluated in testcross trials and 315 DH lines in line per se trials, including lines from cycles C0 to C3, subsets of the C0sel founder lines, and 10 and 14 checks, respectively. Among the checks in the testcross trials were seven commercial hybrids (Table S3).

Field experiments for testcross and line per se performance were conducted in 2023 and 2024 in three or four locations per year, respectively, covering a wide range of agro-climatic conditions in Germany. Trials in each environment were laid out as alpha-lattice designs with two replications and incomplete blocks of 10 plots. The testcross trials were planted in double-row plots 6 m in length, and the line per se trials were planted in single-row plots 3 m in length, with a 0.75 m distance between rows in both cases. Targeted sowing densities followed local practices at the experimental stations and varied between 9 and 12 plants m^−2^. Standard plant protection and fertilization practices were applied. In the testcross trials, biomass yield and dry matter content were assessed. The following traits were measured in both trials (Table S1): final plant height, female flowering, and early plant height in stages V4 and V6.

### Genotypic data

For genotyping the C1-S_1_, C2-S_0_, C3-S_0_, C1-DH, C2-DH, and C3-DH populations, a 15 k SNP custom Illumina® Array developed by KWS SAAT SE & Co. KGaA was used. Processing of the genotypic data, including filtering, quality control, and imputation, followed the protocol detailed in Polzer et al. (2025). After these steps, 11,160 SNP markers (denoted as 11 k SNP array), fully overlapping with the 600 k chip, were retained for further analyses. After quality control and pedigree checks, 1,030 C1-S_1_, 1,003 C2-S_0_, 304 C3-S_0_ genotypes, and 360 DH lines were retained.

### Analyses of phenotypic data

An analysis of variance (ANOVA) was conducted for each lattice design in each environment for filtering raw data using Grubbs’ outlier test (Grubbs 1969). Outliers and plots in line per se trials with fewer than five plants were treated as missing values.

The statistical model for analyzing the phenotypic data from testcross or line per se trials across environments was:

3 $$y_{pikst} = \mu_{p} + g_{i\left( p \right)} + l_{k} + gl_{ik\left( p \right)} + r_{s\left( k \right)} + b_{{t\left( {sk} \right)}} + e_{pikst} ,$$

where ${y}_{pikst}$ is the phenotypic plot-level observation. ${\mu }_{p}$ is the fixed effect of population *p*, where *p* = 1 to 4 refers to the DH populations (C0r, C1-DH, C2-DH, C3-DH), *p* = 5 to the C0sel lines, and *p* = 6 to the checks. ${g}_{i(p)}$ denotes the effect of genotype $i$*,* nested in population *p*. For variance component estimation, ${g}_{i(p)}$ was treated as random for *p* = 1 to 4, allowing for heterogeneous genetic variances ${\sigma }_{g(p)}^{2}$ among populations (${g}_{i(p)}\sim \text{ iid }N(0,{\sigma }_{g(p)}^{2})$), and as fixed for *p* = 5 and 6. Adjusted entry means per genotype were calculated by treating ${g}_{i(p)}$ as fixed. ${l}_{k}$ represents the random effect of environment *k,* and ${gl}_{ik(p)}$ is the random interaction effect for genotype *i* with environment *k*, nested in population *p*, with heterogeneous variances ${\sigma }_{gl(p)}^{2}$ among populations. ${r}_{s(k)}$ and ${b}_{t(sk)}$ are random effects of replication *s* nested in environment *k* and incomplete block *t* nested in replication *s* in environment *k*. ${e}_{pikst}$ is the random residual error of observation ${y}_{pikst}$, with ${e}_{pikst}\boldsymbol{ }\sim \text{ iid }N(0,{\sigma }_{e}^{2})$, and ${\sigma }_{e}^{2}$ denotes the error variance. Adjusted entry means and variance components of random effects were estimated using the restricted maximum likelihood method in the ASReml-R package (Butler et al. 2009). Genotypes with incorrect pedigrees, as determined by marker assays, were treated as checks in all analyses.

### Response to selection

Response to selection was evaluated separately for testcross and line per se performance following the approach of Polzer et al. (2025). Briefly, means (${\mu }_{p},$ *p* = 1–4, Eq. 3) of the DH populations were compared using a global Wald test. If significant, pairwise differences between population means were assessed using Tukey’s honestly significant difference (HSD) test (Tukey 1949). To assess the direct or correlated selection response across cycles for each trait, we performed a linear regression analysis using adjusted entry means of DH lines from the C1-DH to C3-DH populations based on the following model:

4 $$y_{ij} = \beta + \gamma c_{j} + e_{ij}$$

where ${y}_{ij}$ refers to the adjusted entry mean of genotype *i* from cycle *j*. $\beta$ is the intercept; $\gamma$ is the selection response per cycle. The predictor variable ${c}_{j}$ indicates the selection cycle *j*. ${e}_{ij}$ is the residual error, with ${e}_{ij} \boldsymbol{ }\sim \text{ iid }N(0,{\sigma }^{2})$. Selection response was evaluated by testing the following hypotheses: H_0_: $\gamma \le 0$ vs. H_1_: $\gamma > 0$ for biomass yield in testcross performance using a one-sided significance test; H_0_: $\gamma =0$ vs. H_1_: $\gamma \ne 0$ for all other traits in both testcross and line per se performance, using a two-sided test. The same approach was used to evaluate the change in GEBVs per cycle for the DH and the heterozygous populations. In testcross trials, the yield gap between the means of DH populations and the commercial checks was expressed as percentage relative to the mean of the checks.

Heritabilities on an entry-mean basis per cycle were calculated together with their confidence intervals following Hallauer et al. (2010) and Knapp et al. (1985). The variance of GEBVs within each DH and heterozygous population was calculated as the average squared deviations from the respective population mean.

Phenotypic trait correlations were calculated separately for each DH population in the following combinations: (i) between different traits within testcrosses and lines per se, (ii) for the same trait across testcrosses and lines per se, and (iii) between biomass yield in testcrosses and traits in lines per se.

### Prediction accuracy and retraining

As a major factor influencing response to genomic selection, prediction accuracy ($\rho$) was estimated for testcrosses. Prediction ability (${r}_{a}$) was calculated as the correlation between GEBVs and adjusted entry means across environments for the DH population of each cycle (C0r_TP_, C1-DH, C2-DH, C3-DH) evaluated in 2023 and 2024. The estimate of $\rho$ was obtained by dividing ${r}_{a}$ by the square root of the heritability calculated for the entire experiment (Dekkers 2007). GEBVs are calculated using Eq. 1 with PK (N = 419) as training set and subsequently employed to calculate $\rho$. Additionally, $\rho$ for C0r_TP_ was also calculated using a training set, termed PK_no_overlap (N = 388), which excluded founder lines selected from C0 (subset of PK) and DH lines common to PK and C0r_TP_. This adjustment was made to avoid an upward bias in $\rho$ caused by overlap of genotypes between the training and prediction set.

Retraining of the GBLUP model was performed by successively augmenting the original 2018/2019 PK training set with 2023/2024 data from populations C1-DH and C2-DH, thereby generating updated training sets. To enable joint analysis data sets were adjusted to a common mean level using common checks.

## Results

### Response to selection for testcross performance in DH populations

**Fig. 1:**
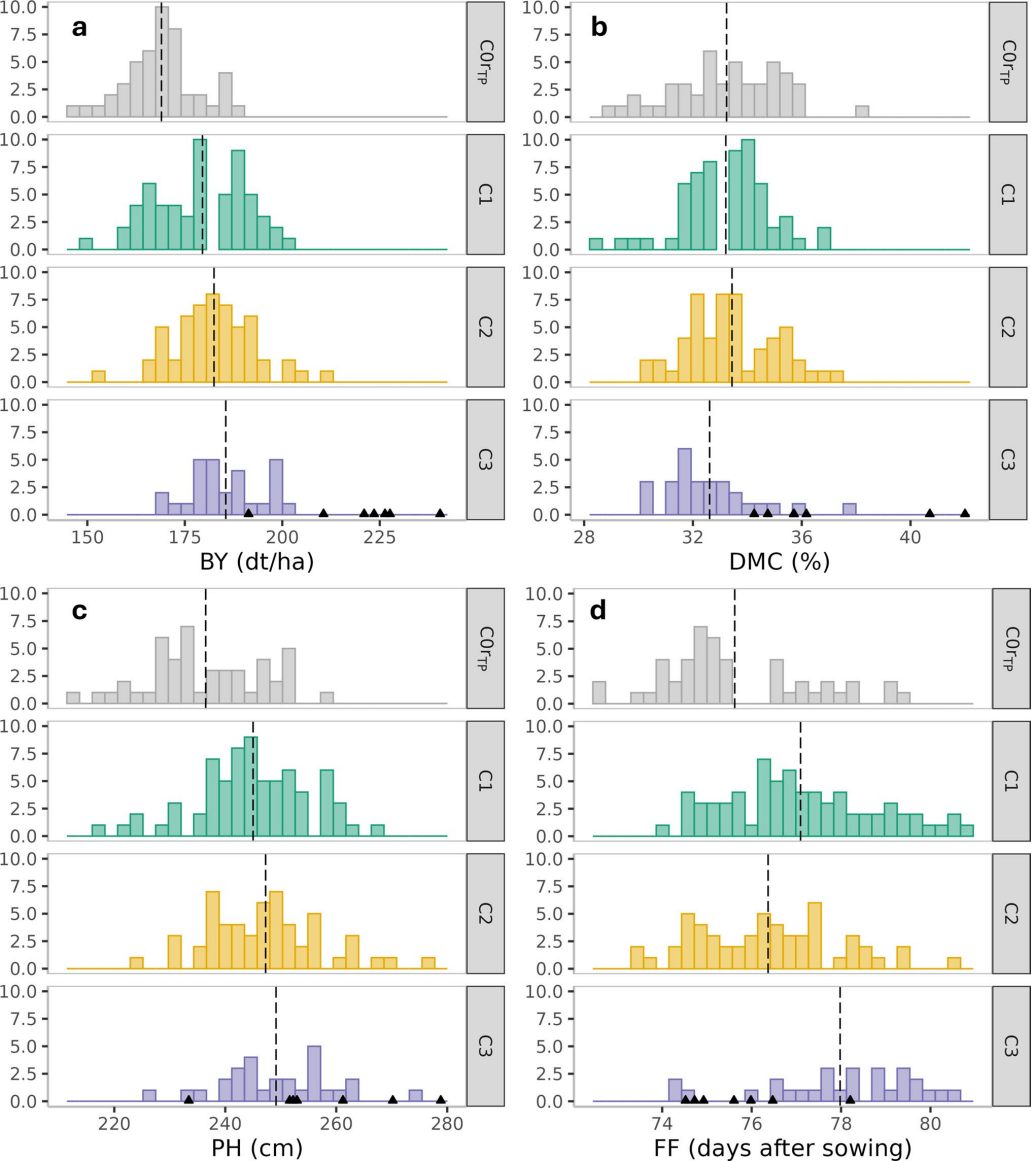
Distributions of adjusted entry means for DH lines from populations C0r_TP_ to C3 for testcross performance of **a** biomass yield (BY), **b** dry matter content (DMC), **c** final plant height (PH), and **d** female flowering (FF). Vertical dashed lines indicate the respective population mean. Triangles along the x-axis indicate the adjusted means of the seven commercial check hybrids

Selection response for testcross performance across cycles was evaluated using adjusted entry means of testcrosses of DH populations derived from cycles C0 to C3 (Fig. 1, Table 1). Biomass yield showed a consistent positive response to selection across cycles, with 6% increase from C0r_TP_ to C1, followed by gains of approximately 2% per cycle from C1 to C3. Although both plant height and female flowering were under stabilizing selection, significant changes in population means could still be observed. A significant increase was observed from C0r_TP_ to C1 for both traits, but no significant increase was observed between C1 and C3. Dry matter content showed no significant correlated response across cycles, only a small decrease in C3 (Fig. 1, Table 1), while plant height in stage V6 increased from C0r_TP_ to C1 but decreased in later cycles (Table S2).

**Table 1:** Means of DH populations from C0r_TP_ to C3 for testcross performance in comparison with seven commercial check hybrids for biomass yield (BY), dry matter content (DMC), final plant height (PH), and female flowering (FF)

	N	BY		DMC	PH	FF
			top 15%			
C0r_TP_	47	169.00^a^	184.45	33.24^a^	236.47^a^	75.62^a^
C1	70	179.53^b^	195.26	33.21^a^	245.01^b^	77.09^bc^
C2	55	182.49^b^	199.57	33.44^a^	247.27^b^	76.37^ab^
C3	28	185.50^b^	198.89	32.61^a^	249.15^b^	77.98^c^
Slope		2.98*	2.26	−0.21	2.10	0.24
Hybrids	7	220.13		37.04	257.21	75.78

Means for biomass yield are also shown for the top 15% of candidates in C0r_TP_ to C3. The linear regression coefficient (Slope) indicates the selection response per cycle from C1 to C3. Different letters indicate statistically significant (*P* < 0.05) pairwise differences between populations. N refers to the number of genotypes

*Significance at the 0.05 level

**Fig. 2:**
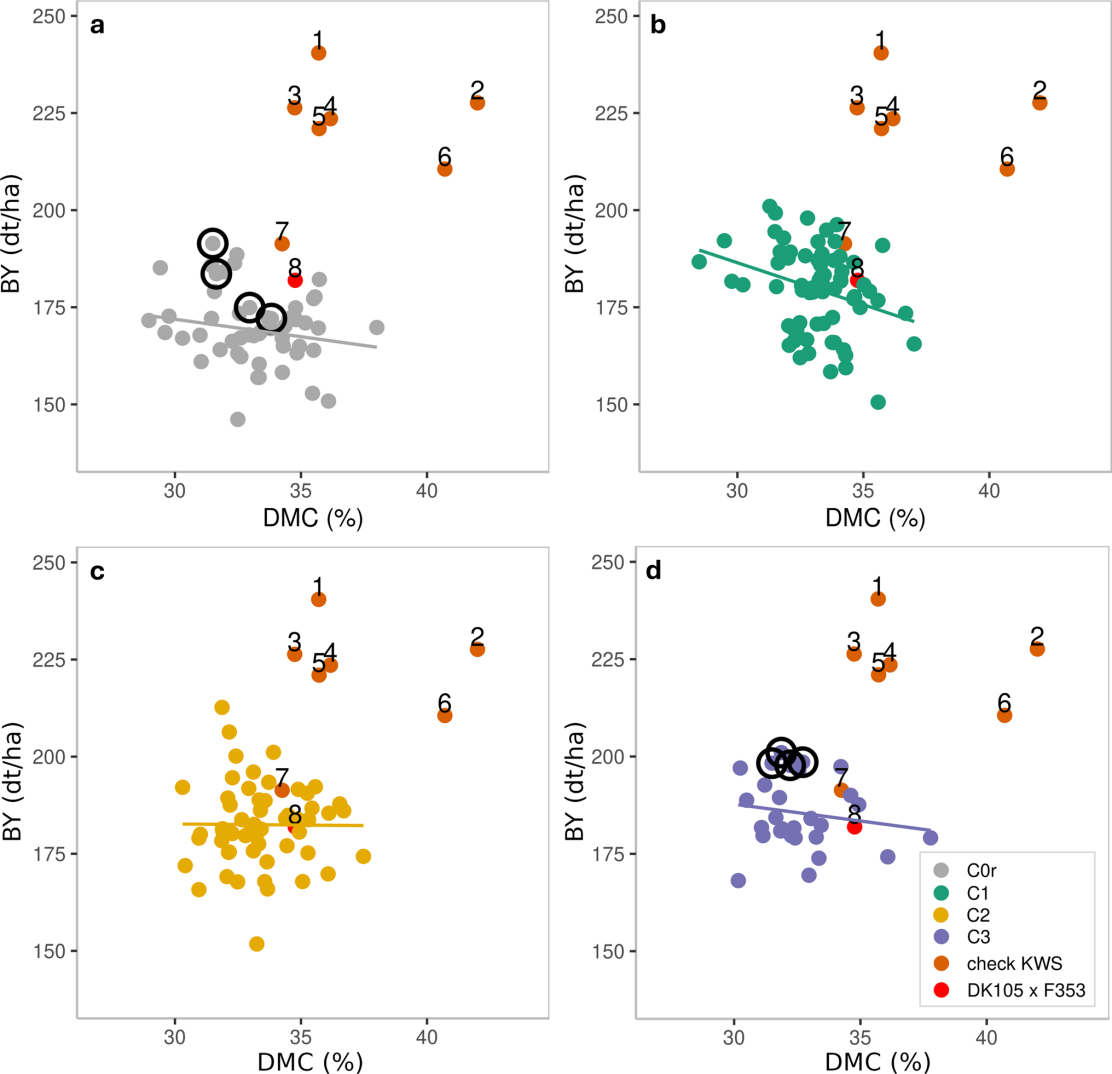
Performance gap between testcrosses of DH lines from cycles **a** C0r_TP_, **b** C1, **c** C2, and **d** C3 with dent tester F353 and checks for biomass yield (BY) and dry matter content (DMC). Checks include hybrid DK105 x F353 (red) and commercial hybrids (brown) developed for silage (1,3), grain (6,7), and dual-purpose use (2,4,5). Black circles indicate selected founder DH lines in C0 and the top 15% performing DH lines in C3

As a result of directional selection for biomass yield, the performance gap between the experimental populations and commercial check hybrids narrowed from 23% in C0r_TP_ to 16% in C3. It is also worth looking at the performance of the best DH lines in cycles C2 and C3. In C2, some of the DH lines reached almost the yield level of some of the checks, but with substantially later maturity (Fig. 2). The population mean for plant height in cycles C2 and C3 remained lower than that of the commercial check hybrids.

Genetic variance estimates (${\sigma }_{g}^{2}$) for biomass yield were comparable between C0r_TP_ and C3 but nearly doubled in cycles C1 and C2 (Table 2). In contrast, ${\sigma }_{g}^{2}$ for the other traits remained relatively stable across cycles, except for a marked reduction for dry matter content when comparing C0r_TP_ and subsequent cycles. Heritabilities ranged from 0.66 to 0.79 for biomass yield and exceeded 0.87 for the other traits in all cycles.

**Table 2:** Genetic variances (with standard errors), and heritabilities (with confidence intervals) in DH populations from C0r_TP_ to C3 for testcross performance of biomass yield (BY), dry matter content (DMC), final plant height (PH), and female flowering (FF)

		BY		DMC		PH		FF	
			Ratio		Ratio		Ratio		Ratio
	C0r_TP_	47.49 ± 16.84	1.00	3.59 ± 0.83	1.00	101.11 ± 23.50	1.00	2.55 ± 0.58	1.00
${\sigma }_{g}^{2}$	C1	86.75 ± 20.04	1.83	2.00 ± 0.40	0.56	95.17 ± 17.80	0.94	2.56 ± 0.48	1.00
	C2	87.01 ± 23.89	1.83	2.34 ± 0.53	0.65	95.59 ± 20.40	0.95	2.37 ± 0.50	0.93
	C3	42.98 ± 22.11	0.91	2.30 ± 0.79	0.64	90.23 ± 28.46	0.89	2.54 ± 0.79	1.00
	C0r_TP_	0.66 (0.61–0.69)		0.92 (0.91–0.93)		0.92 (0.91–0.93)		0.93 (0.92–0.94)	
${h}^{2}$	C1	0.79 (0.76–0.81)		0.89 (0.87–0.90)		0.93 (0.93–0.94)		0.93 (0.93–0.94)	
(CI)	C2	0.74 (0.71–0.77)		0.88 (0.87–0.89)		0.92 (0.91–0.93)		0.92 (0.92–0.93)	
	C3	0.67 (0.61–0.72)		0.87 (0.85–0.89)		0.93 (0.91–0.94)		0.92 (0.91–0.94)	

### Changes in the means of GEBVs in heterozygous and DH populations

Across all cycles, means of GEBVs for biomass yield and plant height showed good concordance between the populations of heterozygous selection candidates (C1-S_1_, C2-S_0_, C3-S_0_) and their corresponding DH populations (Table S4). For these two traits, changes in GEBVs aligned with changes in adjusted means (Table 1). GEBVs for dry matter content were substantially higher in the DH populations compared to the heterozygous populations from which they were derived. These elevated GEBVs in DH lines persisted beyond cycle C1, despite only small changes in adjusted entry means of dry matter content for these populations. For female flowering, GEBVs in cycle C2 decreased in the DH lines, showing the same pattern as the adjusted entry means, as opposed to increased GEBVs in the heterozygous populations.

**Fig. 3:**
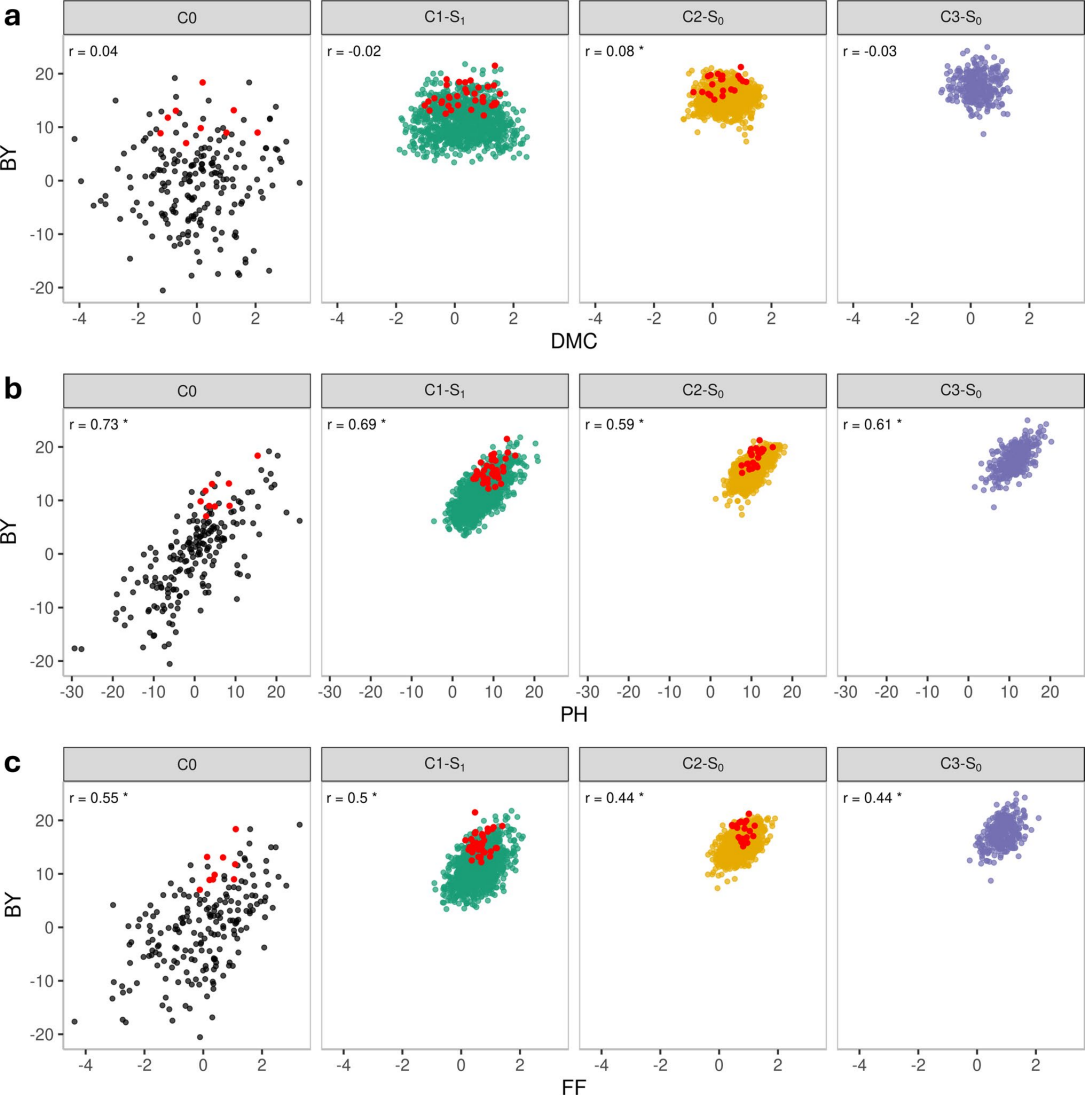
Scatter plot of genomic estimated breeding values (GEBVs) for testcross performance of biomass yield (BY) versus **a** dry matter content (DMC), **b** plant height (PH), and **c** female flowering (FF). Data are shown for DH lines in cycle C0 (N = 203) and heterozygous genotypes in cycles C1 to C3. Selected genotypes in cycles C0 to C2 are highlighted in red. *r* stands for the Pearson correlation coefficient and * indicates significance at the 0.05 level

The influence of the selection criterion on the outcome of selection is evident in the GEBVs of selected individuals (Fig. 3). While selected genotypes had GEBVs for biomass yield that were drawn from the upper half of the population distribution, they did not necessarily represent the highest-ranking individuals. For plant height and female flowering that were under stabilizing selection, selected genotypes tended to have intermediate GEBVs. In contrast, for dry matter content GEBVs of selected genotypes were spread across a broader range.

The variance of GEBVs for DH lines decreased substantially for all traits from C0 to C1 (Fig. S3, Table S5). For biomass yield, dry matter content, and female flowering, an additional decline in GEBV variance was observed after the second selection step, particularly pronounced for biomass yield. The GEBV variance was consistently higher in the fully homozygous DH populations than in the corresponding heterozygous parent populations in all cycles and for all traits. An important factor contributing to differences in the variance of GEBVs between cycles was the level of inbreeding of selection candidates in the populations, with an average inbreeding coefficient of F = 1.0, 0.5, and 0 for C0, C1, and C2, respectively. Changes across cycles in the variance of the selection criterion follow closely those of biomass yield (Table S5). However, the changes across cycles in the variance of GEBVs for DH lines did not always correspond to the changes in ${\sigma }_{g}^{2}$ for the adjusted entry means (Table 2, Table S5). For instance, for biomass yield a decrease in GEBV variance was observed from C0 to C1, while ${\sigma }_{g}^{2}$ for the adjusted entry means increased.

### Molecular differentiation of cycles C0 to C3

**Fig. 4:**
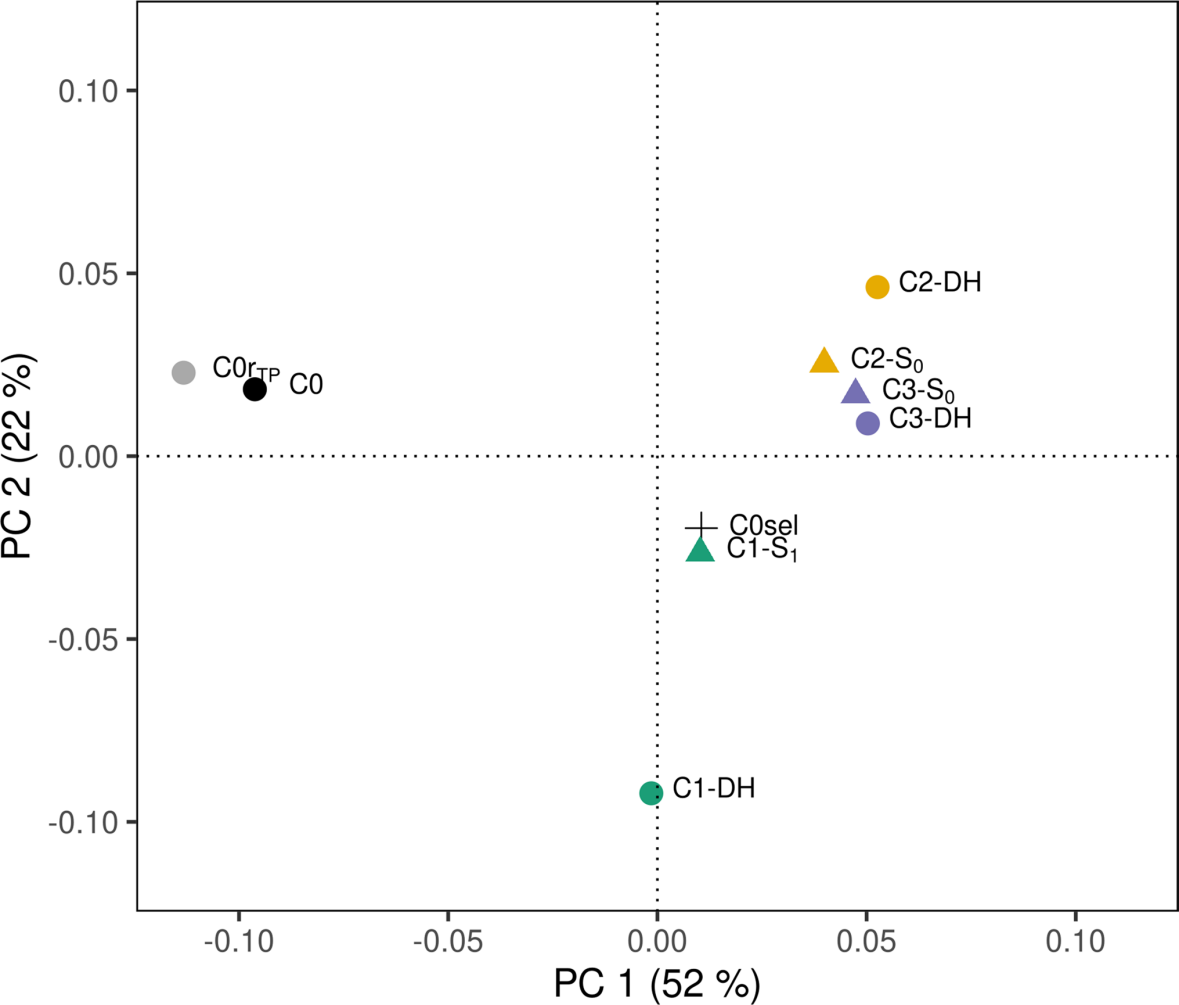
Principal coordinate analysis of eight populations from the selection experiment, as well as C0sel founder lines. Calculations were based on modified Rogers’ distances derived from SNP data. Colors represent selection cycles (C0, C0r_TP_, and C1 to C3), and different symbols differentiate DH lines from S_1_ and S_0_ genotypes

Figure 4 presents the results of a principal coordinate analysis for DH, S_1_, and S_0_ populations from cycles C0 to C3 based on modified Rogers’ distances estimated from the molecular data. The first principal coordinate (PC1) explained 52% of the total variance and clearly separated populations from cycles C0, C1, and C2. The greatest molecular divergence was observed between C0 and C1, consistent with changes of adjusted entry means and GEBVs across cycles. While C2 and C3 populations had similar loadings on PC1, they were separated from C1 along PC2, which accounted for 22% of the total variance. As expected, the selected founder lines (C0sel) clustered with the C1-S_1_ population and the C0r_TP_ population grouped closely to the original C0 (PE), indicating that it represented the original population well at a whole-genome level. In cycles C2 and C3, DH populations clustered closely with their corresponding S_0_ populations, confirming their allelic similarity. In contrast, the DH population in C1 showed quite some divergence along PC2 from the C1-S_1_ population.

### ***Trait correlations for cycles C0r***_***TP***_*** to C3***

**Fig. 5:**
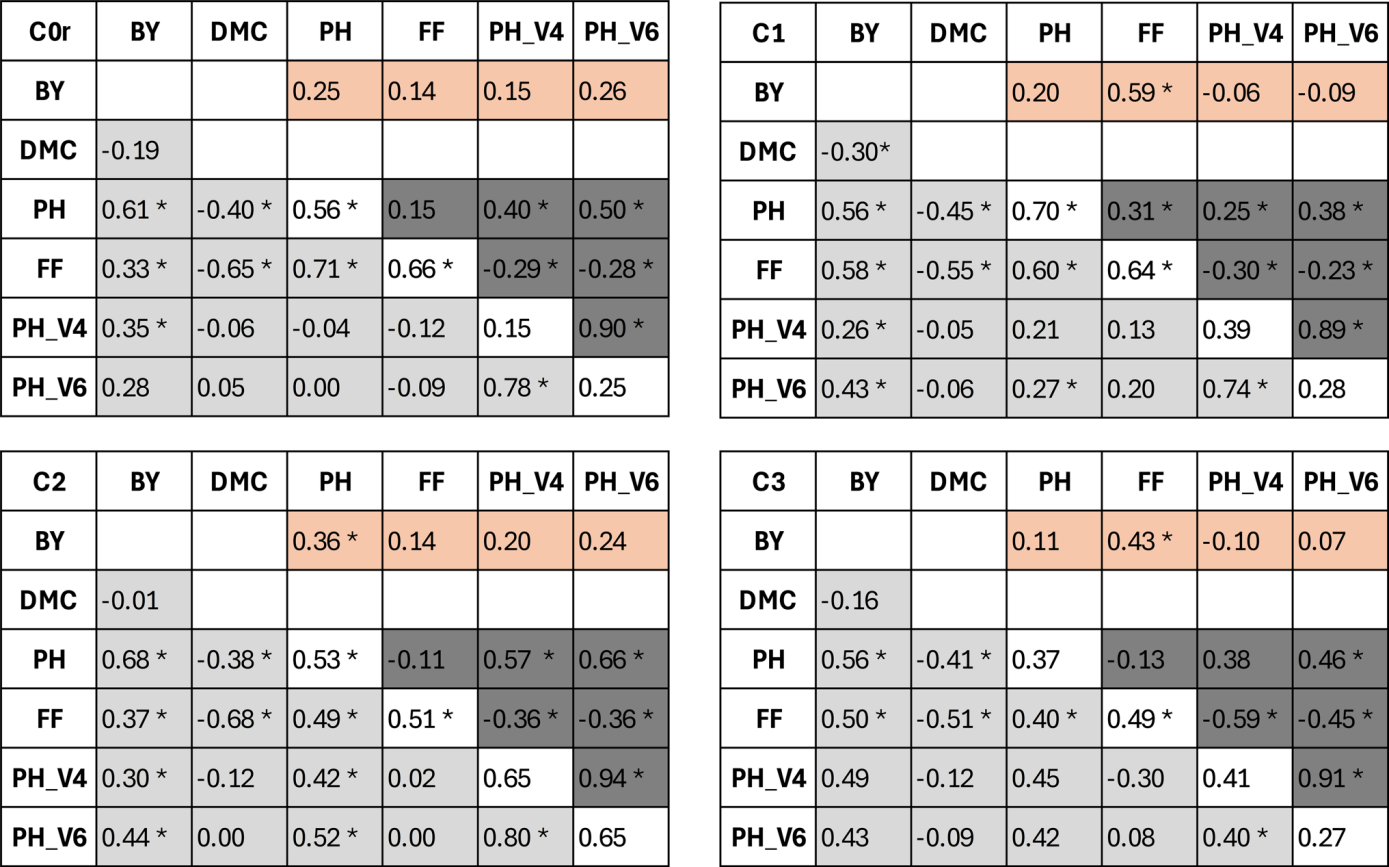
Phenotypic correlations between traits for testcrosses (gray cells below the diagonal) and lines per se (dark gray cells above the diagonal) as well as for the same traits between testcrosses and lines per se (white cells along the diagonal) for selection cycles C0r to C3. Traits include biomass yield (BY), dry matter content (DMC), final plant height (PH), female flowering (FF), and early plant height at growth stages V4 (PH_V4) and V6 (PH_V6). The top row (coral cells) displays correlations between biomass yield (BY) in testcrosses and other traits in lines per se

Phenotypic correlations among the three selected traits (testcross performance for biomass yield, plant height, and female flowering) were significantly positive across cycles C0r_TP_ to C3 and generally of intermediate magnitude (Fig. 5). Dry matter content was negatively correlated with plant height and female flowering in all cycles. There was no significant correlation between biomass yield and dry matter content, except for cycle C1, where the correlation was negative. The correlation between plant height and female flowering was consistently positive and of intermediate magnitude across cycles. Early plant height showed moderate correlation with biomass yield. The correlation with plant height was low in cycles C0 and C1, but increased in later cycles.

### ***Prediction accuracies in DH populations of cycles C0r***_***TP***_*** to C3***

Prediction accuracies ($\rho )$ of GEBVs for traits evaluated in testcrosses ranged from 0.69 to 0.81 in C0r_TP_ with PK as the training set and from 0.53 to 0.71 when excluding DH lines from the training set that overlapped with C0r_TP_ (Table 3). Accuracies declined to intermediate levels in cycle C1 for all traits. For biomass yield and female flowering, $\rho$ dropped further in cycle C2 but increased again in C3, surpassing C1 levels for biomass yield. In contrast, $\rho$ for plant height remained stable across cycles C1 to C3. For dry matter content, $\rho$ progressively declined over cycles, reaching its lowest level (0.10) in cycle C3.

**Table 3:** Prediction accuracies ($\rho$) for testcross performance in prediction sets C0r_TP_ to C3 with and without retraining

Training set	N_TS_	Prediction set	N_PS_	BY	DMC	PH	FF
PK	419	C0r_TP_	47	0.69	0.77	0.75	0.81
PK_no_overlap	388			0.67	0.53	0.62	0.71
PK	419	C1	70	0.40	0.55	0.43	0.47
PK	419	C2	55	0.30	0.40	0.45	0.24
PK + C1	489			0.52	0.39	0.53	0.46
C1	70			0.51	−0.06	0.28	0.38
PK	419	C3	28	0.49	0.10	0.40	0.42
PK + C1	489			0.60	0.09	0.59	0.57
PK + C2	474			0.72	0.33	0.68	0.54
PK + C1 + C2	544			0.66	0.32	0.72	0.61
C1	70			0.38	−0.07	0.40	0.26
C2	55			0.49	0.47	0.66	0.35
C1 + C2	125			0.48	0.47	0.68	0.46

All prediction sets were evaluated in field trials conducted in the years 2023 and 2024. The initial training set (PK) included 419 DH lines from landraces Petkuser and Kemater evaluated for testcross performance in 2018 and 2019. For retraining, DH lines from cycles C1 and C2 tested in 2023 and 2024 were added to the training set. For prediction of C0r_TP_, an additional training set (PK_no_overlap) was used in which all lines overlapping with C0r_TP_ were excluded from PK to avoid data leakage. Traits are biomass yield (BY), dry matter content (DMC), final plant height (PH), and female flowering (FF). N_TS_ and N_PS_ denote the size of the training and prediction set, respectively

The effect of retraining on $\rho$ was evaluated by incorporating DH lines from cycles C1 and C2 into the training set (Table 3). In prediction set C2, $\rho$ values increased substantially when C1 lines were added except for dry matter content. Notably, using C1 alone as training set yielded $\rho$ values comparable to those obtained with the much larger training set PK for biomass yield and female flowering. In cycle C3, retraining with DH lines from both C1 and C2 increased $\rho$ across all traits.

### Response to selection for line per se performance in DH populations

Indirect response to selection across cycles was evaluated for plant height, female flowering, and early plant height in lines per se using adjusted entry means of DH populations from cycles C0 to C3 (Table S2). Within each cycle, about half of the DH lines evaluated for line per se performance overlapped with those assessed for testcross performance. Similar trends in mean differences across cycles were observed in lines per se and in testcrosses for the given traits (Table S2). Correlations between testcross and line per se performance of the same trait were generally positive and of moderate magnitude (Fig. 5). In contrast, correlations between biomass yield in testcrosses and traits measured in lines per se were generally low, except for female flowering in cycles C1 and C3.

## Discussion

### Closing the yield gap between landraces and elite germplasm

In this study, we investigated the potential of rapid cycling genomic selection for reducing the performance gap between landrace-derived and breeding material. Selection was based on GEBVs of DH lines produced from a pre-selected flint landrace and crossed to a dent tester. In contrast to many other studies, that used either progenies from landrace x elite backcrosses or testcrosses of heterogeneous landrace populations (Babic et al. 2022; Pollak 2003; Sanchez et al. 2024), we quantified the yield gap based on testcross performance of DH lines derived directly from the landrace Petkuser Ferdinand Rot. We chose this approach, because it allows to purge the landrace from some of its genetic load (Melchinger et al. 2017) and avoids the loss of beneficial alleles from the donor landrace due to unintentional selection favoring the genome of the elite parent, suggested to be a major drawback of backcrossing programs (Gorjanc et al. 2016; Hölker et al. 2022).

Over the three cycles of selection, we observed a significant increase in biomass yield. In comparison with seven commercial hybrids, the initial average performance gap of the landrace-derived DH lines amounted to 23% in C0, which is similar in magnitude to what was reported for other European flint landraces for silage (Brauner et al. 2019) and grain yield (Böhm et al. 2014; Wilde et al. 2010). While on average the performance gap to the checks was still 16% in C3, some DH lines in C2 performed almost as well as the checks with respect to biomass yield albeit with a lower dry matter content (Fig. 2). This was the case even though the tester was released about 25 years ago and was not selected for specific combining ability with landrace material making these DH lines interesting candidates for crosses with elite material.

It is unrealistic to assume that the performance gap between landraces and elite material generated by decades of intense breeding for many traits can be closed with three cycles of genomic selection. However, with rapid cycling genomic selection we now have the means to accelerate pre-breeding of landraces. Recurrent phenotypic selection using either half-sib or reciprocal full-sib methods typically requires four to five growing seasons—equivalent to two to three years. Hallauer et al. (2010) report selection response for yield to range from 2 to 4% per population and cycle (defined as the time from the initial crosses to the evaluation of the progenies as testcrosses in the field). As maize breeding technology allows three consecutive generations per year, two additional recombination and selection steps (defined as cycles in this study) can be integrated within the same timeframe. We showed that even without retraining about 2% genetic gain can be accomplished in each of these additional cycles. Moreover, the high costs for phenotyping in traditional recurrent selection typically limits population sizes to 100 to 200 families, while genomic selection enables the analysis of a large number of selection candidates (here ~ 1,000 individuals in C1 and C2) allowing for high selection intensities. Therefore, it seems realistic to conclude that rapid cycling genomic selection can increase annual genetic gain significantly in pre-breeding programs. It will also reduce the time span required before the best landrace-derived lines can be submitted to a bridging program, which is recommended as an intermediate step before introducing genetic resources into the elite breeding pool (Allier et al. 2020). However, in this context a crucial question remains: When is the optimal point for transitioning from the pre-breeding to the bridging phase? Future experimental studies should provide guidelines to answer this question. The genetic material developed in this and in our companion study (Polzer et al. 2025) will provide the basis for experiments addressing this topic.

While our results are encouraging, their generality across landraces remains to be shown. For example, Ordás et al. (2023) reported high variation in response to recurrent phenotypic selection for grain yield across four landraces. One reason was that these landraces varied strongly for maturity, genetic diversity, and overall performance level. Nevertheless, the effect of sampling on the outcome of selection experiments can also be seen in our study. The genetic makeup of the high yielding DH lines found in the C2-DH population (Fig. 1) was very likely not represented in C2-S_0_. In addition, the PCoA differentiated C1-DH and C1-S_1_ based on allele frequencies (Fig. 4). When comparing the average GEBVs of the DH populations with their respective parental populations (C1-S_1_, C2-S_0_, C3-S_0_), we observed pronounced differences for dry matter content in all cycles (Table S4) and for female flowering in C2 suggesting unconscious selection for earlier genotypes during DH production. Thus, the effect of random sampling and of unconscious selection for secondary traits not included in the selection criterion must be taken into account when interpreting results of an unreplicated selection experiment. The observed doubling of genetic variance for biomass yield in C1-DH compared to C0r (Table 2) must also be attributed partially to sampling effects. Additionally, the apparent increase in genetic variance may have been enhanced by the family structure created in cycle C1 after crossing the founder lines. Moreover, the elimination of detrimental alleles during selection from C0 to C1 may have released genetic variance at loci that had not contributed to genetic variance in C0.

### Multi-trait selection

Introducing landrace-derived material into elite germplasm can only be successful if it performs satisfactorily for yield and all other agronomically relevant traits. In addition to maximizing yield, correlated response in traits like plant height and female flowering must be avoided and undesirable traits like lodging and disease susceptibility must be eliminated. Consequently, many traits must be improved simultaneously in recurrent selection. When establishing the population to start the selection experiment, a mild selection against DH lines with extreme lodging was applied. Nevertheless, segregation for lodging could still be observed in all selection cycles. Our selection criterion combined directional selection for biomass yield with stabilizing selection for plant height and female flowering. We had chosen these two traits in contrast to dry matter content as in the training population (PK) they had exhibited significant correlations with biomass yield while dry matter content had not (Hölker et al. 2019). This pattern persisted across selection cycles. Despite selection toward the mean for plant height and female flowering, we observed a pronounced increase of both traits from C0 to C1 and a loss of earlier flowering genotypes from C2 to C3 (Table 1, Fig. 1). We conclude that the selection criterion applied in our study might not have been optimal for sufficient control of plant height and female flowering, but a restricted selection index (Kempthorne and Nordskog 1959) or a nonlinear merit function might have been more appropriate, as they enforce zero expected response or penalize deviations from the desired trait values while allowing improvement in yield. For example, Gianola and Fernando (1986) suggested to use Bayesian approaches for finding solutions for complex nonlinear multi-trait selection problems. Irrespective of the statistical approach used, selection for multiple traits requires the evaluation of large numbers of candidates and rapid cycling genomic selection can be part of the solution. Given intermediate prediction accuracies for all traits, rapid cycling genomic selection allows high-throughput screening of heterozygous selection candidates for multiple traits at reasonable costs. How to implement an optimal multi-trait rapid cycling genomic selection scheme for pre-breeding warrants further research. First ideas based on the results of our study can be found in the following section.

### Implementation of rapid cycling genomic selection

Selection response per cycle depends on the selection intensity, the variance of the selection criterion among selection candidates, and the correlation between the selection criterion and the true breeding values (TBVs) in the population. For the latter, prediction accuracy (*ρ*) estimated for the traits included in the selection criterion can be used as a proxy.

In this study, the selection proportion α was about 4% in cycle C0 and about 3% in cycles C1 and C2, resulting in comparable selection intensities (${i}_{\alpha }\cong 2.2)$ across all cycles. However, the effective population size ${N}_{e}$ of the selected fraction varied markedly between cycles, with ${N}_{e}\cong$ 4.5, 27, and 32 in C0, C1, and C2, respectively. Small ${N}_{e}$ in combination with high selection pressure likely accounted for the strong reduction in variance of GEBVs of selection candidates and DH lines in advanced cycles for individual traits and the selection criterion (Table S5). This strong reduction in variance partly explains the decrease in selection response from C1 to C3 as compared to the first selection from C0 to C1.

The second parameter determining the success of genomic selection is the prediction accuracy $\rho$. In C0, the realized $\rho$ can be estimated, because selection was conducted among DH lines phenotyped for testcross performance. In subsequent cycles, the realized $\rho$ refers to the correlation between the GEBVs of the heterozygous selection candidates and their unknown TBVs for testcross performance. This realized $\rho$ can only be inferred by crossing each selection candidate to a tester and evaluating the resulting testcrosses in the field. In an academic program, the required logistics were prohibitive, and therefore, we assessed $\rho$ in random sets of DH lines derived from each cycle (Table 3). This approach assumes that the DH lines are representative samples of the selection candidates and that recombination during DH production has a minimal impact on linkage disequilibrium (LD) between markers and QTL. As discussed above, this assumption might have been violated explaining to some extent the low accuracies for female flowering and dry matter content observed in cycles C2 and C3 in the DH lines, but we need to keep in mind that estimates of $\rho$ have high standard errors, especially in C3 (Polzer et al. 2025).

Estimates of $\rho$ in C0r_TP_ were intermediate to high across all traits which is consistent with results of Hölker et al. (2022), who also reported promising prediction accuracies for testcross performance of DH lines derived from landraces. While $\rho$ decreased slightly in subsequent cycles, it remained at moderate levels for most traits except dry matter content. Retraining the model with genotypes from prior cycles improved $\rho$, with the greatest gains observed when the most recent cycle was added to the training set. These results are consistent with theoretical expectations regarding key determinants of $\rho$ in genomic selection: additive-genetic relationships between the genotypes in the training and prediction sets, co-segregation between markers and QTL, and LD (Habier et al. 2013). The combined effect of these factors is strongest when the training set is genetically similar to the prediction set. Consequently, prediction in C3 profited more from incorporating C2 in the training set than from C1. However, in our experiment the DH lines of all cycles were evaluated in the same environments, which in practice would not be the case. As the difference in accuracy was small when retraining with C1 and C2 as compared to C2 only, it should be possible in pre-breeding to aggregate data sets from different cycles evaluated in different years to capture genotype x environment interactions, which has been shown to improve prediction accuracies substantially in breeding populations (Auinger et al. 2016, 2021).

The retraining strategy must also be balanced against the temporal constraints of rapid cycling, which differ depending on whether selection targets line per se or testcross performance. For traits assessed for line per se performance, phenotyping must be conducted on homozygous lines to obtain GEBVs free from dominance effects. This necessitates the production of inbred lines for model training, as implemented in this study and in our companion study on line per se performance for early development traits (Polzer et al. 2025). The process of generating, multiplying, and phenotyping these fully homozygous lines, e.g. by the DH technology, requires at least three growing seasons. Thus, the phenotypic information available for retraining lags behind the ongoing selection, limiting its immediate impact on selection response.

In contrast, selection for testcross performance enables a more timely and efficient integration of model retraining. Testcross performance can be assessed reliably using both homozygous and heterozygous genotypes, as its underlying genetic architecture can be described by an additive model, even in the presence of dominance (Melchinger et al. 1998). Thus, the heterozygous candidates with the highest GEBVs can serve as pollen parents to produce testcross progenies for phenotyping and simultaneously for recombination. This enables testcross performance evaluation in parallel with genotyping candidates of the next cycle, reducing the time lag in model retraining. Such synchronization supports the central goal of rapid cycling genomic selection: to accelerate pre-breeding in comparison with recurrent phenotypic selection. Moreover, phenotyping the same candidates that are also recombined to produce the next generation ensures a close genetic relationship between the training and prediction sets, which enhances $\rho$ in genomic selection (Habier et al. 2013; Schopp et al. 2017).

Looking ahead, we propose to further explore the potential of rapid cycling genomic selection with an integrated strategy combining line per se and testcross performance. In an academic context, it is impossible to account for all relevant factors contributing to the success of such a program simultaneously. Here, we show the results for one landrace, with one replication and with one inbred tester. Additional selection experiments are needed that can be aggregated in meta-analyses to obtain a more comprehensive picture. Some questions will have to be answered in experimental studies like the variation of results across landraces. Evaluating testcross performance with more than one elite tester also seems advisable to assess general combining ability in different genetic backgrounds. In addition, we consider landraces as valuable sources for novel diversity presumably lost in advanced cycle populations. It will therefore be interesting to evaluate the yield gap to commercial checks under low-input and environmental stress conditions.

Given the substantial time and resource demands of experimental rapid cycling genomic selection, we also advocate for the use of data-based simulations to explore different implementation scenarios. The data generated in this study, in Polzer et al. (2025), and in many of our flanking projects (e.g. Mayer et al. (2022)) offer a robust empirical foundation for converging on realistic assumptions for modeling the molecular and phenotypic variation in landraces. The estimated quantitative genetic parameters will guide us when exploring major design factors of rapid cycling genomic selection such as intermating schemes, effective population size, and selection intensity. Simulations will also allow to explore different overarching goals for pre-breeding as demonstrated by Bernardo (2009, 2016). If the aim is to rapidly generate lines as parents in bridging programs, a high selection intensity is justified. Conversely, for long-term improvement strategies—similar to those used in traditional phenotypic recurrent selection (Hallauer 1992)—one might favor milder selection pressure to maintain genetic diversity. Considering the strong reduction in variances of GEBVs in our study, despite restricting the number of progenies per founder line (Table S5), we will explore if a less stringent selection in C0 followed by increased selection pressure in advanced cycles may be advantageous.

Despite all these possibilities for further research, our results provide valuable insights with respect to implementing rapid cycling genomic selection to close the performance gap in general combining ability for yield and agronomically important traits between landraces and elite material. As scientists from one of the most comprehensive genebanks worldwide stated “*there is an urgent need for better ways to […] realize the evolutionary potential of large seed collections locked away in cold rooms*” (Mascher et al. 2019). The continued breeding progress in elite germplasm will widen the gap making it progressively more difficult to use these materials for coping with future challenges in crop production. Thus, if we don’t do it now, then when?

## Conclusions

We demonstrated that rapid cycling genomic selection is a highly effective strategy for accelerating pre-breeding in landraces and narrowing the performance gap between landrace-derived and elite breeding material. Our study offers initial guidelines for designing multi-trait selection experiments aimed at this objective. Integrating DH production with rapid cycling genomic selection provides a powerful approach to unlocking the untapped genetic variation conserved in thousands of accessions in genebanks. Nevertheless, several key questions remain, including the optimal selection intensity over cycles and the most effective intermating schemes to promote recombination. Further research is also warranted to develop strategies for efficiently introducing selections from landraces into elite breeding programs. The insights from this study lay the ground for future experiments and simulation-based research to address these challenges.

## Supplementary Information

Below is the link to the electronic supplementary material.

Supplementary file1 (DOCX 745 KB)

## Data Availability

All data and material are available through material transfer agreements upon request.
