# Supplementary material for: Rapid cycling genomic selection in maize landraces: a step toward closing the yield gap
Source: Theor Appl Genet. 2025 Dec 15;139(1):5. doi: 10.1007/s00122-025-05107-3 (PMC12705729; doi:10.1007/s00122-025-05107-3)
Supplement: Supplementary file 1 — Supplementary file1 (DOCX 745 KB) [file 122_2025_5107_MOESM1_ESM.docx]

**Rapid cycling genomic selection in maize landraces: a step towards closing the yield gap**

Carolina Rivera-Poulsen^1c^, Clara Polzer^1,b,c^, Armin C. Hölker^1,a^, Thomas Presterl^2^, Sofia da Silva^2^, Michelle Terán-Pineda^1^, Milena Ouzunova^2^, Albrecht E. Melchinger^1,3^, Chris-Carolin Schön^*1^

^1^ Plant Breeding, TUM School of Life Sciences, Technical University of Munich, Freising, Germany, 85354

^2^ KWS SAAT SE & Co. KGaA, Einbeck, Germany, 37574

^3^ Institute of Plant Breeding, Seed Science and Population Genetics, University of Hohenheim, Stuttgart, Germany, 70593

^a^ present address: KWS SAAT SE & Co. KGaA, 37574 Einbeck, Germany

^b^ present address: HZPC Research B.V., Roptawei 4, 9123 JB Metslawier, The Netherlands

^c^ these authors contributed equally to this work

^*^Corresponding author

Email address and telephone number:

chris.schoen@tum.de, +498161713422

**Supplementary figures**

**
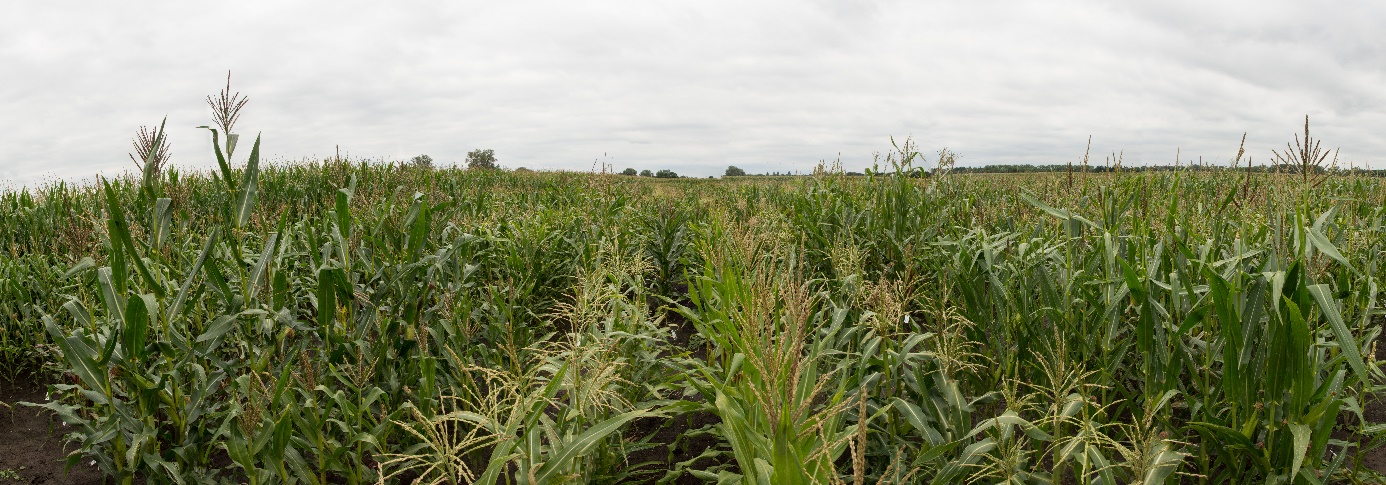
**

**Figure S1** Field evaluation of DH lines derived from landraces Petkuser Ferdinand Rot (PE) and Kemater Landmais Gelb (KE)


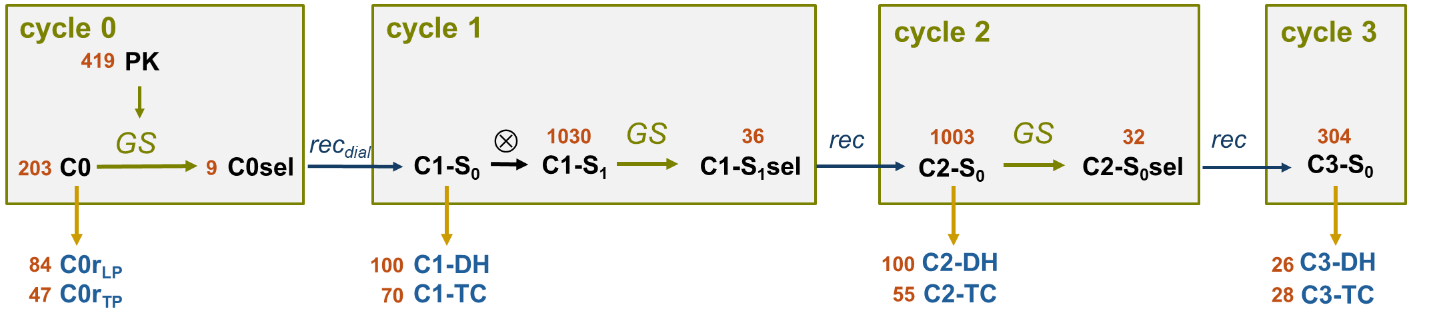


**Figure S2** Rapid cycling genomic selection scheme. Selection in all cycles was based on a multi-trait selection criterion on testcross performance with training set PK (N = 419). The best C0sel lines from the C0 DH population (PE) were selected, recombined in a diallel crossing scheme and selfed once. Genomic selection and mating of selected candidates in pairs was repeated for two consecutive cycles. From each cycle DH lines and their testcrosses were produced and evaluated in field trials. Scheme modified from Polzer et al. (2025)


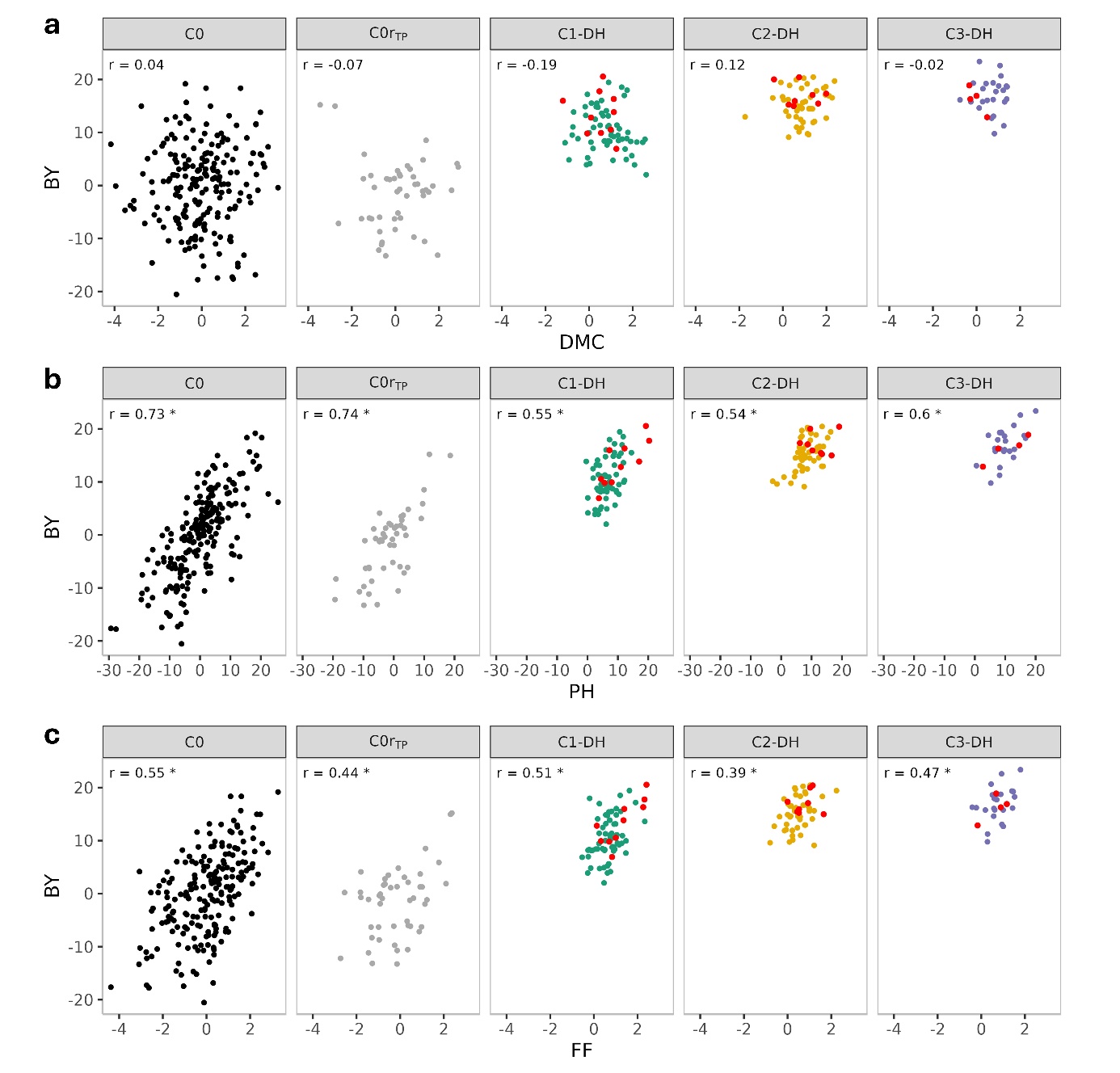


**Figure S3** Scatter plot of genomic estimated breeding values (GEBVs) for testcross performance of biomass yield (BY) versus **(a)** dry matter content (DMC), **(b)** plant height (PH), and **(c)** female flowering (FF). Data are shown for DH populations C0, C0r_TP_ and C1 to C3. The top 15% DH lines for biomass yield (BY) in cycles C1 to C3 are highlighted in red. r stands for the Pearson correlation coefficient and * indicates significance at the 0.05 level

**Supplementary tables**

**Table S1** Traits, abbreviations, description of trait collection and environments of testcross (TP) and line per se (LP) trials for each trait

| **Trait** | **Abbreviation** | **Description** | **Environments** | |
| --- | --- | --- | --- | --- |
|  |  |  | **TP** | **LP** |
| Biomass yield | BY | Total dry matter yield inferred from total amount of biomass per plot adjusted to the % of dry matter content. | Roggenstein 2023/24 Grucking 2023/24 Klein Wanzleben 2023/24 Einbeck 2024 |  |
| Dry matter content | DMC | Dry matter content inferred through drying of a sample. | Roggenstein 2023/24 Grucking 2023/24 Klein Wanzleben 2023/24 Einbeck 2024 |  |
| Final plant height | PH | In cm, from ground to first branch of the tassel. Average over three plants. | Roggenstein 2023/24 Grucking 2023/24 Klein Wanzleben 2023/24 Einbeck 2024 | Roggenstein 2024 Einbeck 2023/24 Bernburg 2023/24 Oberer Lindenhof 2023 |
| Days to female flowering | FF | Days after sowing when 50% of the plants have fully developed female flowers (upper 1-2 cm of the silk visible). | Roggenstein 2023/24 Grucking 2023/24 Klein Wanzleben 2023/24 Einbeck 2024 | Roggenstein 2024 Einbeck 2023/24 Bernburg 2023/24 Oberer Lindenhof 2023 |
| Early plant height | PH_V4 and PH_V6 | In cm, from ground to longest, manually vertically streched leaf, in stages V4 and V6. Average over three plants. | Roggenstein 2024 Grucking 2024 Klein Wanzleben 2024 Einbeck 2024 | Roggenstein 2024 Einbeck 2023/24 Bernburg 2023/24 Oberer Lindenhof 2023 |

**Table S2** Means of DH populations C0r to C3 and seven commercial check hybrids for testcross (TP) and line per se performance (LP) of early plant height in stages V4 and V6 (PH_V4, PH_V6), final plant height (PH) and female flowering (FF). The linear regression coefficient (Slope) indicates the correlated selection response per cycle from cycle C1 to C3. Different letters indicate statistically significant (*P<0.05*) differences between populations. N_TP_, N_LP_ and N_∩_ refer to the number of genotypes for TP, LP and their overlap, respectively

|  |  |  |  | **TP** | | **LP** | | | |
| --- | --- | --- | --- | --- | --- | --- | --- | --- | --- |
|  | **N_TP_** | **N_LP_** | **N_∩_** | **PH_V4** | **PH_V6** | **PH_V4** | **PH_V6** | **PH** | **FF** |
| **C0r_TP/LP_** | 47 | 84 | 34 | 53.25 ^a^ | 127.80 ^a^ | 42.06 ^a^ | 84.18 ^a^ | 131.60 ^a^ | 80.78 ^a^ |
| **C1** | 70 | 100 | 55 | 54.35 ^a^ | 131.25 ^b^ | 44.06 ^a^ | 90.12 ^b^ | 141.00 ^a^ | 83.10 ^b^ |
| **C2** | 55 | 100 | 41 | 54.60 ^a^ | 130.89 ^ab^ | 42.13 ^a^ | 85.75 ^ab^ | 137.96 ^a^ | 82.19 ^ab^ |
| **C3** | 28 | 26 | 25 | 53.23 ^a^ | 128.81 ^ab^ | 40.60 ^a^ | 83.47 ^ab^ | 142.35 ^a^ | 85.30 ^b^ |
| **Slope** |  |  |  | -0.32 | -0.97 | -1.79 * | -3.66 * | -0.50 | 0.50 |
| **Hybrids** | 7 |  |  | 50.54 | 123.91 |  |  |  |  |

* Significance at the 0.05 level

**Table S3** Commercial check hybrids (KWS SAAT SE & Co. KGaA) included in the evaluation trials, with their primary end use

|  | **Hybrid name** | **End use** |
| --- | --- | --- |
| **1** | Kilomeris | Silage |
| **2** | Amarola | Dual-purpose |
| **3** | Walterinio KWS | Silage |
| **4** | KWS Editio | Dual-purpose |
| **5** | Figaro | Dual-purpose |
| **6** | KWS Stabil | Grain |
| **7** | KWS Gustavius | Grain |

**Table S4** Means of genomic estimated breeding values (GEBVs) for testcross performance of DH populations C0r_TP_ to C3, selected founder lines (C0sel), population C0 (PE), and heterozygous populations (C1-S_1_, C2-S_0_, and C3-S_0_) for biomass yield (BY), dry matter content (DMC), final plant height (PH), and female flowering (FF). The linear regression coefficient (Slope) indicates the change in GEBVs per cycle from cycle C1 to C3 for S_1_/S_0_ and DH populations

|  | **BY** | **DMC** | **PH** | **FF** |
| --- | --- | --- | --- | --- |
| **C0** | 0 | 0 | 0 | 0 |
| **C0r_TP_** | -1.66 | 0.12 | -1.63 | -0.16 |
| **C0sel** | 11.11 | 0.15 | 5.78 | 0.54 |
| **C1-S_1_** | 11.27 | 0.34 | 6.97 | 0.60 |
| **C1-DH** | 10.83 | 0.74 | 7.05 | 0.74 |
| **C2-S_0_** | 15.37 | 0.48 | 9.65 | 0.69 |
| **C2-DH** | 15.43 | 0.89 | 8.75 | 0.52 |
| **C3-S_0_** | 17.62 | 0.24 | 11.04 | 0.86 |
| **C3-DH** | 16.90 | 0.58 | 10.09 | 0.76 |
| **Slope S_1_/ S_0_** | 3.43 * | 0.01 | 2.21 * | 0.12 * |
| **Slope DH** | 3.31 * | -0.04 | 1.53 * | -0.03 |

**Table S5** Variances of genomic estimated breeding values (GEBVs) for testcross performance of DH populations C0r_TP_ to C3, the population C0 (PE), and heterozygous populations (C1-S_1_, C2-S_0_, C3-S_0_) for biomass yield (BY), dry matter content (DMC), final plant height (PH), and female flowering (FF), and for the selection criterion (SC). GEBVs were calculated using the training set PK (N = 419). Ratios represent the proportion of GEBV variance of each population relative to the C0 population

|  | **BY** | | **DMC** | | **PH** | | **FF** | | **SC** | |
| --- | --- | --- | --- | --- | --- | --- | --- | --- | --- | --- |
|  |  | **ratio** |  | **ratio** |  | **ratio** |  | **ratio** |  | **ratio** |
| **C0** | 61.78 | 1.00 | 2.01 | 1.00 | 78.98 | 1.00 | 1.85 | 1.00 | 3.14 | 1.00 |
| **C0r_TP_** | 42.34 | 0.69 | 1.89 | 0.94 | 53.56 | 0.68 | 1.47 | 0.79 | 1.77 | 0.56 |
| **C1-S_1_** | 10.75 | 0.17 | 0.71 | 0.35 | 15.47 | 0.20 | 0.26 | 0.14 | 0.38 | 0.12 |
| **C1-DH** | 17.45 | 0.28 | 0.76 | 0.38 | 17.14 | 0.22 | 0.41 | 0.22 | 0.63 | 0.20 |
| **C2-S_0_** | 4.11 | 0.07 | 0.29 | 0.14 | 6.31 | 0.08 | 0.13 | 0.07 | 0.24 | 0.08 |
| **C2-DH** | 8.90 | 0.14 | 0.57 | 0.28 | 17.70 | 0.22 | 0.30 | 0.16 | 0.57 | 0.18 |
| **C3-S_0_** | 6.08 | 0.10 | 0.22 | 0.11 | 9.63 | 0.12 | 0.15 | 0.08 | 0.33 | 0.11 |
| **C3-DH** | 9.86 | 0.16 | 0.37 | 0.18 | 20.80 | 0.26 | 0.30 | 0.16 | 0.42 | 0.13 |
